# Structural, Optical, and Neutron Sensitivity Properties of Yb^3+^/Tb^3+^ Codoped Alkaline Earth Tetraborates

**DOI:** 10.1021/acsomega.5c04342

**Published:** 2025-09-10

**Authors:** Özde Ceren Hızal, Okan Esenturk, Damla Çetin Altındal, Ayşen Yılmaz

**Affiliations:** a Department of Chemistry, Middle East Technical University, Ankara 06531, Turkey; b Department of Micro and Nano Tech., Middle East Technical University, Ankara 06531, Turkey; c Department of Bioengineering, 37515 Hacettepe University, Beytepe,Ankara 06800, Turkey

## Abstract

This study explores the synthesis, structural properties, and multifunctional potential of Yb^3+^/Tb^3+^ codoped alkaline earth tetraborates (CaB_4_O_7_, MgB_4_O_7_, and SrB_4_O_7_) synthesized via solid-state (SS), solution combustion (SC), and combustion (C) methods. The multifunctional potential of the synthesized materials arises from their unique combination of properties in one material. The first property is related to their high boron content and thus to their high neutron capture cross section, providing effective functionality for boron neutron capture therapy (BNCT) and shielding. This enables the particles to be selective tumor cell destruction agents under neutron irradiation. The second property is their strong upconversion luminescence that allows for near-infrared light-triggered photodynamic therapy. This offers deep-tissue treatment capability along with BCNT. The third property is their excellent photochemical stability and low biotoxicity. This combination of therapeutic modalities within a single platform holds promise for synergistic and minimally invasive cancer treatment strategies, along with imaging capabilities and drug delivery. X-ray diffraction analysis confirmed the phase purity and crystal structures of CaB_4_O_7_ (CBO), MgB_4_O_7_ (MBO), and SrB_4_O_7_ (SBO). CBO crystallizes in a monoclinic structure with a *P*2_1_/*n*(14) space group, while both MBO and SBO adopt orthorhombic structures with *Pcba* and *Pnm*2_1_ space groups, respectively. Scanning electron microscopy revealed particle sizes ranging from 100 to 200 nm, with more uniform and smaller particles achieved by SC. Strong upconversion luminescence, driven by cooperative energy transfer between Yb^3+^ and Tb^3+^ ions, was observed in all materials, particularly in CBO and MBO codoped with 5% Yb^3+^ and 5% Tb^3+^. The quantum yields (QY) for CBO and MBO reached approximately 0.35. Furthermore, neutron sensitivity was significantly enhanced with ^10^B isotope enrichment, making these materials promising candidates for BNCT and neutron shielding applications. Cytotoxicity tests confirmed the biocompatibility of the materials, especially for CBO and MBO, which have maintained high cell viability.

## Graphical abstract

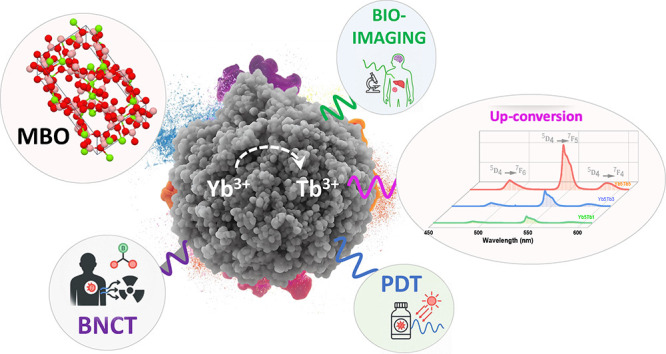

## Introduction

Borates, particularly those doped with rare-earth elements, have attracted significant attention in chemistry and material sciences. Their structural and optical properties, particularly when doped with ytterbium (Yb^3+^) and terbium (Tb^3+^), have shown potential for innovative applications in fields of photonics, biotechnology, and energy conversion.

The versatility of borates is demonstrated by their potential for applications in many studies in diverse technological and medical fields. For instance, the studies investigated such materials for applications in medicine for imaging and targeted drug delivery,^1^ in material engineering for development of smart materials,^2–4^ and in optics.^5,6^ Additionally, they serve as critical components in electronics as a cathode or insulating materials,^7,8^ in chemistry for enhancing catalytic activities,^4,9^ in energy for enhancing solar absorption^2^, and in environmental science for pollutant degradation^10^.

Among the various multifunctional materials, the boron-containing compounds stand out due to their promising roles in nonlinear optics, photonics, and cancer treatments. These materials are particularly useful in photodynamic therapy (PDT) and boron neutron capture therapy (BNCT), where their high boron content and biocompatibility make them ideal candidates for enhanced therapeutic efficacy. High-density boron particles doped with luminescent agents allow real-time monitoring of boron biodistribution while also offering potential for neutron shielding applications. However, despite these promising uses, the combination of rare-earth codoping (e.g., Yb^3+^/Tb^3+^) in borate compounds remains underexplored, especially in the context of upconversion and neutron capture.

Boron-based compounds doped with lanthanide ions (e.g., Yb, Tb, and Er) exhibit strong luminescent radiation, well-defined emission spectra with significant anti-Stokes shifts, and prolonged excited-state lifetimes. Their sharp f–f electronic transitions enable efficient and tunable emissions spanning the ultraviolet (UV), visible, and near-infrared (NIR) regions. Luminescence in these spectral ranges plays a pivotal role in a variety of scientific and technological applications, including bioimaging, optical sensing, photovoltaics, laser materials, and photodynamics.^11,12^ These properties, along with high photochemical stability and low biotoxicity,^13–15^ make these materials highly suitable for applications such as PDT, bioimaging, sensing, and energy conversion. Although borates,^16^ oxides,^17^ and fluorides^18^ have been extensively studied, research on the Yb^3+^ and Tb^3+^ codoped borates remains limited. This is particularly intriguing given the potential of these materials for cooperative energy transfer (CET),^14,19–21^ a mechanism that offers enhanced optical properties ideal for applications in biotechnology and photonics.

Studies on borates mainly focused on Nd^3+^, Tb^3+^, Sm^3+^, Gd^3+^, or Dy^3+^ singly doped borates such as CaB_4_O_7_,^22–26^ MgB_4_O_7_,^27^ and SrB_4_O_7_.^28–30^ In addition, other studies have explored upconversion materials utilizing Er^3+^, Ho^3+^, Tm^3+^, or Eu^3+^ ions codoped with Yb^3+^.^28,29^ Very few studies, on the other hand, have explored the codoping of Yb^3+^ and Tb^3+^ ions in nonborate compounds.^30,31^ Thus, the promising combination of codoped CaB_4_O_7_, MgB_4_O_7_, or SrB_4_O_7_ by Yb^3+^ and Tb^3+^ ions remains unexplored.

Yb^3+^/Tb^3+^ codoped alkaline earth tetraborate compounds are especially promising for their strong upconversion emissions and multifunctionality. In addition to their optical properties, borates are widely utilized in BNCT due to their high neutron capture cross section, nonradioactivity, and biocompatibility. Historically, boron agents such as sodium borocaptate (BSH) and boronophenylalanine (BPA) have been used in clinical applications^32^ but limitations in their therapeutic effectiveness have driven the search for developing novel boron carriers. Borates and doped materials with a high boron ratio represent the next generation of boron agents, offering increased efficacy and greater versatility.^33–37^

Our primary aim is to evaluate the structural, optical, and functional properties of these Yb^3+^/Tb^3+^ codoped tetraborates to assess their suitability as multifunctional platforms for applications in boron neutron capture therapy (BNCT), photodynamic therapy (PDT), and potential biomedical imaging. In this study, we synthesized high-density boron-containing Yb^3+^/Tb^3+^ codoped tetraborates of calcium (CaB_4_O_7_), magnesium (MgB_4_O_7_), and strontium (SrB_4_O_7_) using three distinct synthesis techniques. Three distinct synthesis methods—solid-state (SS), solution combustion (SC), and combustion (C)—were systematically investigated to determine the most effective synthesis method for minimizing secondary phase formation, achieving uniform dopant distribution for enhancing upconversion emission and reducing particle size for improved material performance. The SC method involves dissolving all precursors in an aqueous medium prior to the combustion reaction, allowing homogeneous mixing at the molecular level. This often results in improved phase purity and more uniform dopant distribution. In contrast, the C method utilizes solid-phase mixing of precursors, which can lead to less uniformity in the final product due to incomplete mixing. Although both the C and SS synthesis methods employ solid precursors, the SS method lacks the rapid exothermic reaction characteristic of combustion, leading to slower particle formation, larger particle sizes, and less control over dopant dispersion. In this study, all three methods were systematically evaluated to investigate how differences in the reaction mechanisms and energy input affect the structural and optical properties of the synthesized materials. The synthesis and characterization of these compounds were conducted to explore their upconversion properties, neutron sensitivities, and biocompatibility. This work represents one of the first comprehensive studies on Yb^3+^/Tb^3+^ codoped tetraborates with promising implications for their use in BNCT and other advanced medical and technological applications.

## Materials and Methods

### Materials

All precursors were used with analytical purity. NH_4_NO_3_, urea, Sr­(NO_3_)_2_, H_3_BO_3_, CaCO_3_, MgCO_3_, SrCO_3_ (Merck, with 99% purity), Mg­(NO_3_)_2_, and Ca­(NO_3_)_2_ (Sigma, with 99% purity) were used as precursors. Nitrates of the dopants were prepared from their oxides Yb_2_O_3_ (Sigma, with 99% purity, and Tb_4_O_7_, Alfa Aesar, 99.9% purity) by treating them in HNO_3_ (Merck, with 99% purity) solution. As the Boron-10 isotope source, H_3_^10^BO_3_ (ChemCruz, 98% purity) was used.

### Selection of High-Density Boron Agents

In BNCT, the high uptake of the ^10^B isotope and the ability to image this uptake are crucial. Studies highlighted the need for a large number of boron atoms per cell for successful treatment. Yanagië et al.^38^ estimated 11 × 10^9^ boron atoms per cell with BSH, a common BNCT molecule, while Ramachandran et al.^39^ reported around 1500 boron atoms in 4 nm B_2_O_3_ particles. Although boron compounds of group II appear to be a good source of boron, they vary in phase and boron density; thus, selecting efficient BNCT systems requires hosts with a high boron content. The selected borates synthesized in this study—CaB_4_O_7_ (CBO), MgB_4_O_7_ (MBO), and SrB_4_O_7_ (SBO)—offer significantly higher boron densities than many other borates, as shown in Table 1.

**Table 1:** Number of Boron Atoms in Various Borate Compounds

compound	structure	space groups	number of boron atoms per unit cell	estimated number of boron atoms in 10 nm spherical particle^a^
CaB_2_O_4_	orthorhombic	*Pbcn*	8	13.6 × 10^3^
**CaB_4_O_7_**	**monoclinic**	*P*2_1_/*n*(14)	**32**	**17.7 × 10^3^**
Ca_2_B_2_O_5_	monoclinic	*P*12_1_/*c*1	8	9.4 × 10^3^
Ca_2_B_6_O_11_	monoclinic	*P*12_1_/*c*1	24	16.5 × 10^3^
Ca_3_(BO_3_)_2_	trigonal	*R*3*c*	4	8.0 × 10^3^
**MgB_4_O_7_**	**orthorhombic**	*Pcba*	**32**	**17.0 × 10^3^**
Mg_2_B_2_O_5_	monoclinic	*P*12_1_/*c*1	8	11.8 × 10^3^
Mg_3_(BO_3_)_2_	orthorhombic	*Pnnm*	4	9.9 × 10^3^
SrB_2_O_4_	cubic	Pa3	24	15.5 × 10^3^
**SrB_4_O_7_**	**orthorhombic**	Pnm2_1_	**36**	**16.9 × 10^3^**
Sr_3_(BO_3_)_2_	trigonal	*R*3*c*	4	6.8 × 10^3^

a Estimated number of boron atoms in 10 nm spherical particles from the number of boron atoms in their unit cells.

### Synthesis of Yb^3+^ and Tb^3+^ Codoped CBO, MBO, and SBO

MB_4_O_7_ (M: Ca, Mg, and Sr) products were synthesized via solid-state (SS), solution combustion (SC), and combustion (C) synthesis methods. Synthesis parameters indicated in Table 2 were determined for hosts after optimizations of synthesis methods.

**Table 2:** Calcination Temperature and Time of MB_4_O_7_ (M: Ca, Mg, and Sr)

	CaB_4_O_7_	MgB_4_O_7_	SrB_4_O_7_
SS	850 °C, 2 h	800 °C, 2 h	850 °C, 2 h
SC	800 °C, 2 h	850 °C, 2 h	900 °C, 4 h
C	850 °C, 2 h	800 °C, 2 h	900 °C, 4 h

#### Solid-State Synthesis Method

Stoichiometric amounts of MCO_3_ (M: Ca, Mg, and Sr), H_3_BO_3_ (or H_3_^10^BO_3_) with 1:4 mol ratios, and the oxides of Yb^3+^ and Tb^3+^ ions were mixed thoroughly in an agate mortar and transferred to a porcelain crucible. Then, the mixture was heated to 850 °C (800 °C for MgB_4_O_7_) with a 4 °C/min heating rate, with 2 h of calcination time at this temperature. Expected reaction mechanisms are as follows:

$$H_{3}{\mathrm{BO}}_{3}⇌\alpha -{\mathrm{HBO}}_{2}+H_{2}O(170\;{}^{\circ}C)$$

$$2(\alpha -{\mathrm{HBO}}_{2})⇌B_{2}O_{3}+H_{2}O(250\;{}^{\circ}C)$$

$${\mathrm{MCO}}_{3}+2B_{2}O_{3}\to {\mathrm{MB}}_{4}O_{7}+{\mathrm{CO}}_{2}↑(M:\mathrm{Ca},\mathrm{Mg},\;\mathrm{and}\;\mathrm{Sr})$$

#### Solution Combustion Synthesis Method

A mixture of M­(NO_3_)_2_ (M: Ca, Mg, and Sr):H_3_BO_3_:NH_4_NO_3_:urea with 1:4:5:5 molar ratios was prepared. Dopants were added as nitrates of Yb^3+^ and Tb^3+^ prepared from their oxides. With respect to the calculated doping percentage, the required amount of Yb­(NO_3_)_3_ was prepared by dissolving Yb_2_O_3_ with nitric acid (10 mL) in a 1:2 molar ratio. Once a clear solution was obtained, the nitric acid was slowly evaporated. All precursors were dissolved in deionized water and placed in a porcelain crucible. Then, water was removed by heating the solution slowly while continuously mixing and placing it into a preheated muffle oven at 580 °C for 20 min. After combustion was completed, the product was calcined at 800, 850, or 900 °C depending on the host, as referred to in Table 2 for 2 h with a 4 °C/min heating rate.

#### Combustion Synthesis Method

A mixture of M­(NO_3_)_2_ (M: Ca, Mg, and Sr):H_3_BO_3_:NH_4_NO_3_:urea with 1:4:5:5 molar concentrations was thoroughly mixed in an agate mortar and placed in a porcelain crucible. Nitrate forms of dopants were used. Rapid combustion was provided by placing the mixture into a preheated muffle oven up to 580 °C for 20 min. Then, the mixture was calcined for 2 h at 850 °C for CaB_4_O_7_, 2 h at 800 °C for MgB_4_O_7_, and 4 h at 900 °C for SrB_4_O_7_.

$$H_{3}{\mathrm{BO}}_{3}⇌\mathrm{\alpha ‐}{\mathrm{HBO}}_{2}+H_{2}O\;(170\;{}^{\circ}C)$$

$$2(\mathrm{\alpha ‐}{\mathrm{HBO}}_{2})⇌B_{2}O_{3}+H_{2}O\;(250\;{}^{\circ}C)$$

$$M({\mathrm{NO}}_{3}{)}_{2}+2B_{2}O_{3}\to {\mathrm{MB}}_{4}O_{7}+3{\mathrm{NO}}_{2}↑\;(M:\mathrm{Ca},\mathrm{Mg},\mathrm{Sr})$$

### Instruments and Parameters

The XRD patterns were recorded with a Rigaku Miniflex X-ray Diffractometer CuKα (30 kV, 15 mA, and 1.54051 Å) with a 1°/min rate. SEM images were taken via an FEI Quanta 200 FEG ESEM instrument with an Everhart–Thornley detector. High vacuum mode at 5–15 kV was used. Upconversion emission measurements were carried out using a home-built setup with 980 nm continuous-wave laser excitation. Samples were irradiated with a 77 mW excitation power on an approximately 1 mm^2^ area. The laser intensity was adjusted by a variable neutral density filter and measured with a power meter (Thorlabs). Emission signals were collected through a 720 nm short-pass filter (Thorlabs), focused to the entrance slit of a monochromator (Andor Shamrock 500) with an i2CCD camera (Andor Newton). All measurements were performed on as-synthesized samples without any postsynthesis annealing. Background spectra were recorded prior to each measurement, with the laser beam blocked. Quantum yield (QY) analyses were done with an Edinburgh FS5 spectrofluorometer using an integrating sphere with a power of 1 W and power density of 15 mW/mm^2^ at 980 nm. Neutron sensitivity measurements were taken in a neutron facility. Without any special preparation, the powder samples were compressed into a sample holder to ensure a uniform thickness. An empty sample holder was also measured as a reference during the measurements. An Am-241/Be Isotropic Neutron Source, thermal neutrons with a neutron flux of 3.13 × 10^4^ neutron·cm^2^·s^–1^, was used. Thermal neutron activation analysis was conducted using the ^115^In­(*n*,*γ*)^116m^In reaction as a monitor. The gamma spectra of activated ^116m^In were recorded, and peak areas at 1293.56, 1097.33, and 416.86 keV were measured. Measurements were performed with both the direct thermal neutron beam (I0) and the beam attenuated by the target sample (*I*(*x*)). The thermal neutron macroscopic absorption cross section (Σ_T_) was calculated using the equation *I*(*x*) = I0*e* ^(−Σ_T_ *x*)^, where *x* is the sample thickness (0.2 cm).

### In Vitro Cytotoxicity Study Conditions

The biocompatibility of the synthesized particles at three concentrations (125, 250, and 500 μg/mL) was tested on human umbilical vein endothelial cells (HUVECs). Before application, the particles were used in powder form and sterilized under ultraviolet (UV) light exposure for 1 h to ensure aseptic conditions. HUVECs were subcultured in an RPMI-1640 medium with 10% FBS and 1% penicillin–streptomycin, incubated at 37 °C with 5% CO_2_ (Heraus Instruments, Germany). Upon reaching approximately 80–90% confluency, the cells were detached from the culture flasks using a 0.25% trypsin-EDTA solution. The cell suspension was centrifuged at 1000 rpm for 5 min to pellet the cells. Following centrifugation, the supernatant was discarded and the cell pellet was resuspended in a fresh complete medium. Cells were then counted and seeded into 96-well tissue culture plates at a density of 10^4^ cells/well. After an initial incubation period of 24 h to allow for cell attachment and recovery, the UV-sterilized particles were added to the wells at final concentrations of 125, 250, and 500 μg/mL. Wells containing cells without any particle treatment served as the control group. Subsequent analyses were conducted to evaluate the cytocompatibility of the particles under these conditions.

Cell viability was assessed using the MTT assay after 48 h. The medium was replaced with 200 μL of MTT solution (2.5 mg/mL in PBS) and incubated for 3 h. After removing the medium, 200 μL of DMSO was added to dissolve the formazan, and absorbance was measured at 570 nm with a reference at 690 nm (ASYS Hitech UVM 340 Plate Reader). Viability was calculated with eq 1, with experiments conducted in triplicate.

$$\mathrm{viability}=\left(\frac{A_{\mathrm{treated}}}{A_{\mathrm{control}}}\right)\times 100\%$$ 1

Statistical analysis was performed using a *t* test (GraphPad InStat), with *p* < 0.05, *p* < 0.01, and *p* < 0.001 indicating statistically significant, very significant, and extremely significant differences, respectively.

## Results and Discussion

### Synthesis and Structural Analysis

The hosts CaB_4_O_7_ (CBO), MgB_4_O_7_ (MBO), and SrB_4_O_7_ (SBO) and Yb^3+^/Tb^3+^ codoped tetraborates were synthesized via solid-state (SS), solution combustion (SC), and combustion (C) methods. All measurements were conducted on as-synthesized powder samples with no additional annealing applied.

The XRD patterns of the hosts confirm the formation of the desired phases with the crystal patterns matching the corresponding crystal systems across all the synthesis methods for CBO, MBO, and SBO (Figures S1–S3). For CBO (Figure 1a), the SC and C methods yielded a purer monoclinic phase with minimal secondary phases when codoped. In contrast, the SS method resulted in additional side phases in minor quantities, such as YbBO_3_ and TbBO_3_, at higher doping levels, marked with stars (*) in Figure 1a. Figure 1b shows that the SC method successfully produced orthorhombic MBO, while SS and C had additional side phases, mainly YbBO_3_ and TbBO_3_. For SBO (Figure 1c), the orthorhombic phase was achieved across all synthesis methods without significant side phases, although the SC method provided better phase purity.

**Figure 1:**
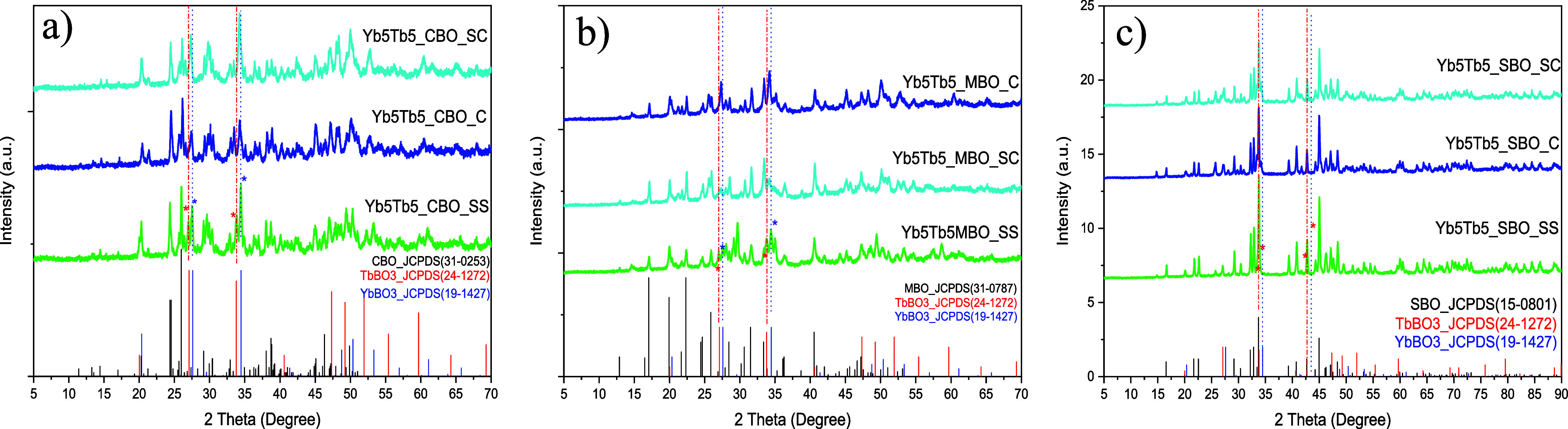
XRD patterns of Yb^3+^/Tb^3+^ codoped (a) CBO, (b) MBO, and (c) SBO obtained via SS, SC, and C methods.

These structural findings suggest that the SC method is the most efficient for achieving high-purity Yb^3+^/Tb^3+^ codoped borates. Table 3 provides a comparison of the lattice parameters and cell volumes, highlighting the impact of Yb^3+^/Tb^3+^ codoping. The Ca^2+^ cations in CBO are coordinated by seven O^2–^ anions within a square-face bicapped trigonal prism environment, which is linked to polyhedrons of (BO_3_)^3–^ and (BO_4_)^4–^ groups. When doped, the trivalent rare-earth ions are expected to be positioned within the tetrahedral sites of the CaB_4_O_7_ structure,^22,40^ which are typically occupied by Ca^2+^ ions. Thus, the SC and C methods led to an expected decrease in cell volume due to the smaller ionic radii of Yb^3+^ (0.925 Å) and Tb^3+^ (0.98 Å) compared to Ca^2+^ (1.106 Å)^41^. However, in SS synthesis, an increase in cell volume was observed unexpectedly, suggesting ineffective doping, possibly due to the placement of a vacant site rather than true ion replacement along with phase formations.

**Table 3:** Unit Cell Parameters of As-Synthesized Undoped and Yb^3+^/Tb^3+^ Codoped MBO, CBO, and SBO samples

	structure, space group		*a* (Å)	*b* (Å)	*c* (Å)	*V*_cell_ (Å^3^)
CBO	monoclinic, *P*2_1_/*n*(14)	YbTb-CBO-SC	12.437	9.843	7.758	949.7
		CBO-SC	12.446	9.859	7.779	954.5
		YbTb-CBO-C	12.423	9.768	7.803	946.9
		CBO-C	12.405	9.883	7.761	951.5
		YbTb-CBO-SS	12.427	9.919	7.801	961.6
		CBO-SS	12.434	9.870	7.770	953.6
MBO	orthorhombic, *Pcba*	YbTb-MBO-SC	8.683	13.668	7.912	939.0
		MBO-SC	8.645	13.649	7.914	933.8
		YbTb-MBO-C	8.591	13.690	7.912	930.6
		MBO-C	8.591	13.613	7.894	923.2
		YbTb-MBO-SS	8.624	13.674	7.937	936.0
		MBO-SS	8.643	13.705	7.939	940.4
SBO	orthorhombic, *Pnm*2_1_	YbTb-SBO-SC	4.428	10.680	4.228	199.9
		SBO-SC	4.426	10.670	4.225	199.5
		YbTb-SBO-C	4.428	10.695	4.232	200.4
		SBO-C	4.425	10.660	4.224	199.3
		YbTb-SBO-SS	4.427	10.680	4.228	199.9
		SBO-SS	4.426	10.667	4.226	199.5

In MBO, Mg^2+^ cations are coordinated by five O^2–^ anions within a trigonal bipyramid environment, which is linked to polyhedrons of (BO_3_)^3–^ and (BO_4_)^4–^. Similarly, the SC and C methods led to an increase in cell volume when Mg^2+^ (0.72 Å) ions were displaced by Yb^3+^ (0.868 Å) and Tb^3+^ (0.923)^41^ while the SS method displayed an unexpected decrease. This may indicate inefficient doping. This was also supported by the formation of Yb and Tb borates during SS synthesis. SBO, in contrast, exhibited no significant change in cell volume, suggesting inefficient doping due to the large size discrepancy between Sr^2+^ (1.31 Å) and the dopant ions {*r*(Yb^3+^) = 1.042 Å and *r*(Tb^3+^) = 1.095 Å at CN = 9}.^2,41^ This limited structural change likely contributed to the weaker optical properties discussed later.

### Morphological Analysis via SEM

The morphology and particle sizes of the synthesized tetraborates were characterized by SEM (Figure 2). Clear differences in particle size and surface structure were observed depending on the synthesis method. Agglomeration was observed for all synthesis methods due to the high synthesis temperature. For CBO, the SS method produced larger, smooth, and spherical particles, whereas the SC and C methods favor the formation of fractured particles, with average sizes below 150 nm. These smaller particle sizes are advantageous for applications that require pass-through cell barriers^42^ or require high surface areas. On the other hand, all methods produced SBO particles with a highly fractured, porous structure, with particle sizes below 100 nm for the C method.

**Figure 2:**
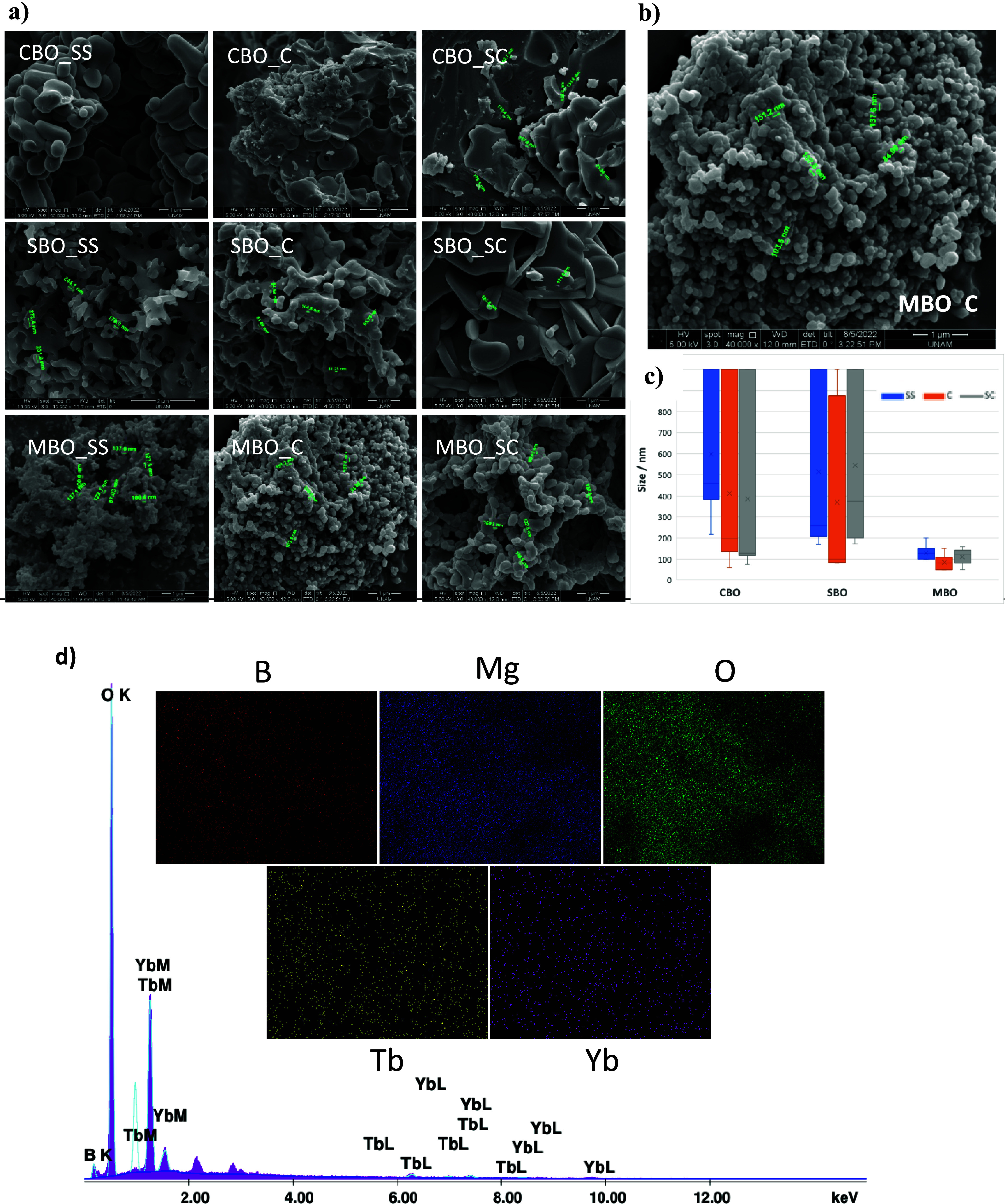
(a) SEM images of CBO, SBO, and MBO crystals synthesized via SS, C, and SC methods, (b) enlarged version of MBO_C, and (c) size distribution of particles less than 1 μm. (d) EDX and mapping of MBO_C.

MBO particles exhibited more uniform morphology across all synthesis methods, producing smooth and spherical particles with minimal agglomeration, corresponding to a relatively lower degree of particle clustering compared to SBO and CBO. The C method yielded the most uniform particles, with sizes generally below 150 nm (Figure 2b), confirming its suitability for MBO synthesis. These SEM results support the idea that the SC and C methods are more effective for producing smaller, uniform particles with favorable surface characteristics, particularly for CBO and SBO. The C method yielded particles below 200 nm for SBO and below 150 nm for MBO.

The particle size distribution histograms of the submicron products synthesized via different methods are presented in Figure 2c. In comparison to SBO and CBO, MBO particles demonstrate significantly smaller and more uniform size distribution, indicating a more controlled morphology under the applied three synthesis conditions. Energy-dispersive X-ray spectroscopy (EDX) and elemental mapping analyses of the Yb^3+^/Tb^3+^ codoped MBO particles (Figure 2d) reveal a uniform distribution of the dopants, with no evidence of elemental impurities. Similar measurements were also performed for the other samples, and comparable homogeneity was observed.

### Upconversion Properties and Cooperative Energy Transfer (CET)

The upconversion emissions of the Yb^3+^/Tb^3+^ codoped tetraborates (Figures 3 and 4) were recorded under 980 nm laser excitation using as-synthesized powder samples, without any annealing treatment. The results demonstrate strong upconversion emissions that are characteristic transitions of the Tb^3+^ ions^43^ in all three hosts, with significant variations in intensity depending on the synthesis method and the host material.

**Figure 3:**
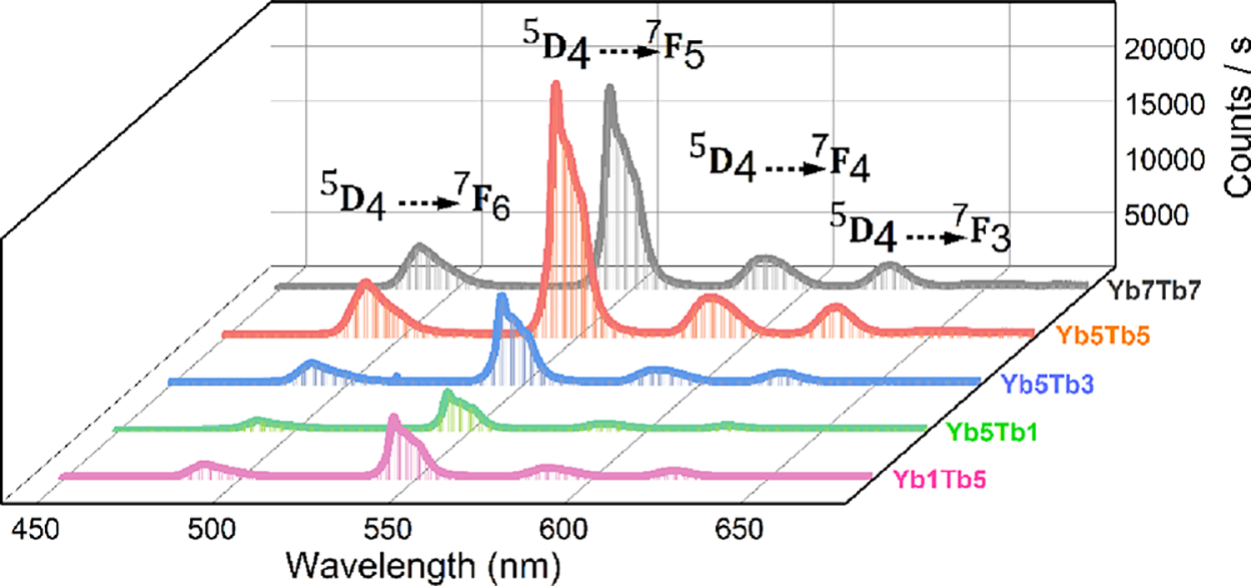
Upconversion emissions of Yb^3+^/Tb^3+^ codoped CBOs prepared by the SC method at 980 nm excitation with a power density of ∼25 mW/mm^2^.

**Figure 4:**
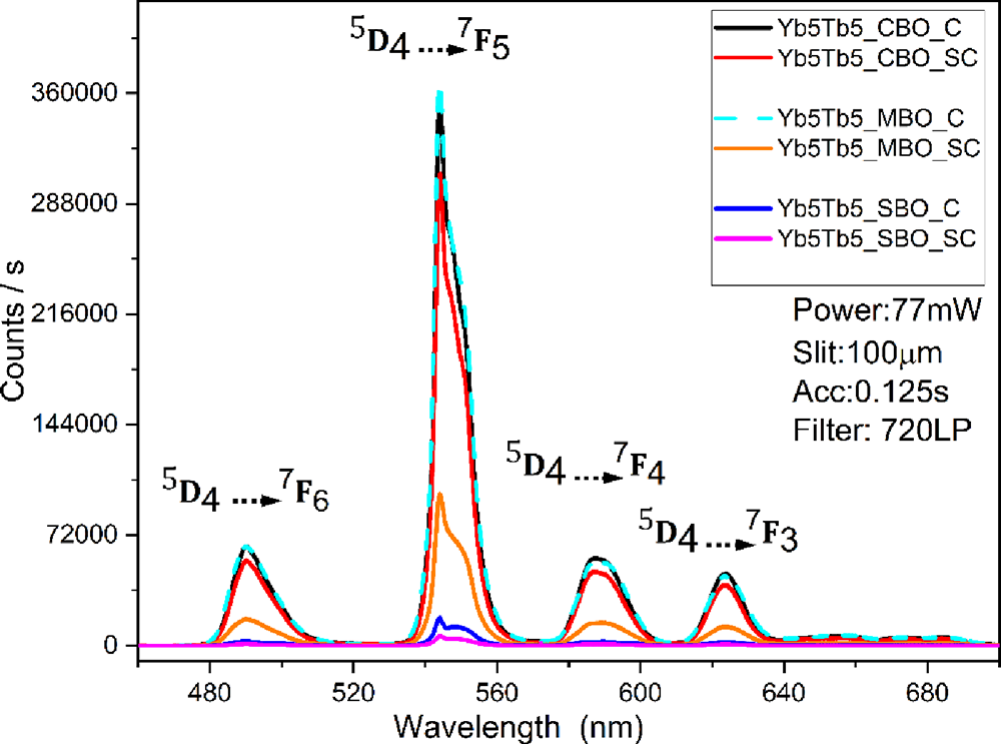
Upconversion emissions of Yb^3+^/Tb^3+^ CBO, SBO, and MBO synthesized by C and SC methods at 980 nm excitation with a power density of 25 mW/mm^2^.

In this study, a range of Yb^3+^ and Tb^3+^ dopant concentrations was systematically investigated to determine the optimal combination for efficient energy transfer and enhanced upconversion emission. Preliminary trials with low doping levels (<1%) were conducted; however, the resulting emissions were too weak to be of practical importance and therefore not included in this work. To optimize both structure and optical performance, dopant concentrations were gradually increased to monitor their influence on crystal phase formation and the emission intensity. Particular attention was paid to avoiding formation of secondary phases that could disrupt the host crystal structure and to staying below the concentration quenching threshold that could reduce emission intensity.

To this end, two sets of samples were prepared. In the first set, the Yb^3+^ concentration was fixed at 5%, and the Tb^3+^ concentration was varied (1, 3, 5, and 7%). In the second set, the Tb^3+^ concentration was fixed at 5%, and the Yb^3+^ concentration was varied from 1 to 5%. Since the upconversion mechanism relies on energy transfer from Yb^3+^ to Tb^3+^ ions under 980 nm excitation, singly Tb^3+^-doped tetraborates were also synthesized to confirm the absence of direct excitation. As shown in Figure S4, no emissions were observed from the singly Tb^3+^-doped or undoped samples under 980 nm excitation, validating that the observed emissions originate from cross-relaxation energy transfer (CET) between Yb^3+^ and Tb^3+^ ions.

Furthermore, to explore the impact of the synthesis method on the optical properties, Yb^3+^/Tb^3+^ codoped tetraborates with selected concentrations were prepared using three different synthesis methods (SS, SC, and C). Samples synthesized via the SC and C methods exhibited significant upconversion emissions, while those synthesized by the SS method did not show notable emission (Figures S5–S7). Therefore, singly doped Tb^3+^ samples were not synthesized by the SS method. Additionally, possible emissions from a potential secondary phase, YbBO_3_, were examined under 980 nm excitation but no detectable signal was observed.

CBO, synthesized via SC, exhibited the strongest upconversion emissions (Figures 3 and 4), with optimal emission intensities observed at Yb5Tb5 (5% Yb^3+^ and 5% Tb^3+^). Emission intensity increased as the Tb^3+^ ion concentration was raised from 1 to 5% but decreased at higher doping levels due to phase formations, as confirmed by the presence of YbBO_3_ and TbBO_3_ in the XRD patterns. These phase formations likely decreased the level of doping and disrupted the cooperative energy transfer (CET) mechanism between Yb^3+^ and Tb^3+^, reducing emission efficiency. Meanwhile, an increase in the Yb^3+^ ion concentration from 1% (Yb1Tb5) to 5% (Yb5Tb5) showed a significant increase in emissions, confirming a strong CET between Yb^3+^ and Tb^3+^ ions. Although the primary factor responsible for the reduction in upconversion emission at high dopant concentrations appears to be the formation of secondary phases associated with the dopants, concentration quenching mechanisms may also contribute to the observed decrease in the luminescence. In these systems, the dominant quenching pathway is likely cross-relaxation between the adjacent Tb^3+^ ions, which becomes significant at elevated concentrations (e.g., 7%), leading to nonradiative energy loss through ion–ion energy transfer. At higher Yb^3+^ doping levels, energy migration among Yb^3+^ ions followed by trapping at defects or other nonradiative centers may further suppress emission. Although back energy transfer from excited Tb^3+^ ions to Yb^3+^ ions is generally considered less probable at moderate concentrations, it may become relevant at high Yb^3+^ levels, potentially resulting in increased Yb^3+^ emission.

A direct comparison of upconversion emissions from codoped CBO, MBO, and SBO (Figure 4) revealed that CBO synthesized via SC and C methods exhibited the highest upconversion efficiencies, with MBO following closely behind. For the MBO case, the structure is known to be suitable for substitution even for large dopant ions.^44–46^ and showed its favored responses. In contrast, SBO exhibited the weakest emissions across all methods, confirming the inefficient doping suggested by XRD, where minimal changes in cell volume were observed and suggested poor incorporation of the dopant ions into the SrB_4_O_7_ lattice. Consequently, the CET mechanism was less effective in SBO than in CBO or MBO. The strong correlation between upconversion intensity and changes in cell volume supports the conclusion that effective displacement doping is the key to enhancing CET and optical performance. This constitutes the first reported upconversion emissions of Tb^3+^ ions in alkaline earth tetraborate hosts, MB_4_O_7_ (M: Ca, Mg, and Sr), where the CET^47,48^ was observed to be very sensitive and significantly impacted by the hosts, the synthesis methods, and the relative dopant amounts.

### Quantum Yield Measurements

The quantum yield (QY) of the codoped CBO and MBO with 5% Yb^3+^ and 5% Tb^3+^ samples upon 980 nm excitation was calculated at approximately 0.35, while the QY for SBO was notably lower at around 0.10 (see Figures S8–S10 for the collected spectra). These results highlight the significant advantage of CBO and MBO for upconversion efficiency, likely due to their more effective doping and structural compatibility with the dopants, as evidenced by the cell volume changes in Table 3.

The lower QY for SBO aligns with its weaker upconversion emissions, further confirming that doping inefficiencies in SBO limit its ability to facilitate effective CET. The QY values observed for CBO and MBO are comparable to those reported in the literature^48–50^ for similar upconversion systems, which typically range between 0.04 and 2% under comparable excitation power densities. Such properties may position these materials as promising candidates for applications requiring high-efficiency upconversion properties, such as bioimaging and sensing.

### Neutron Sensitivity and Boron Isotope Enrichment

The neutron absorption properties of the codoped tetraborates were evaluated to explore their potential for boron neutron capture therapy (BNCT) and neutron shielding applications. Table 4 presents the macroscopic cross sections (Σ_T_) of CBO, SBO, and MBO synthesized with natural ^11^B (19.8% ^10^B isotope) and enriched ^10^B isotopes (98%). Here, the macroscopic cross section represents the effective target area of all of nuclei within the material volume and defines the probability of neutron–nucleus interactions per unit path length of the neutron travel, expressed in units of cm^–1^. Significant increases in neutron absorption were observed with the incorporation of 98% of the ^10^B isotope in all three tetraborates, with CBO showing the highest absorption coefficient (5.85 cm^–1^, a 62% increase). Similar trends were observed for SBO and MBO with 76 and 71% increases, respectively, confirming that enriched borates can significantly improve neutron sensitivity.

**Table 4:** Macroscopic Cross Sections, Σ_T_, of CBO, SBO, and MBO^a^

Σ_T_ (cm^–1^)					
CBO	C^10^BO	SBO	S^10^BO	MBO	M^10^BO
3.62	5.85	3.11	5.48	3.21	5.48

a An empty sample holder was measured under the same conditions as reference. The sample thickness was 0.2 cm.

These findings suggest that Yb^3+^/Tb^3+^ codoped borates, particularly those synthesized with a high ^10^B content, have strong potential as neutron absorbers in BNCT and shielding applications.^51,52^ The high boron content and excellent neutron absorption properties, combined with strong optical emissions, make these materials multifunctional candidates for medical and industrial applications.

### Biocompatibility and Cytotoxicity

In this study, the samples used for biocompatibility and cytotoxicity assessment were Yb^3+^/Tb^3+^ codoped tetraborate particles, synthesized with doping concentrations of 5% Yb^3+^ and 5% Tb^3+^. The biocompatibility of the codoped tetraborates was assessed using MTT assays on HUVEC cells, with particle concentrations of 125, 250, and 500 μg/mL (Figure 5). At a lower concentration (125 μg/mL), all samples showed high cell viability (>80%), indicating good biocompatibility. At higher concentrations (250 and 500 μg/mL), cell viability decreased, particularly for MBO and SBO, although it remained above 60%, suggesting moderate cytotoxicity at these levels. Notably, CBO maintained high biocompatibility even at the highest concentration with a cell viability above 80%. These results, combined with the strong upconversion emissions and neutron sensitivity, position CBO as a particularly promising candidate for biomedical applications, such as imaging and neutron-based therapies.

**Figure 5:**
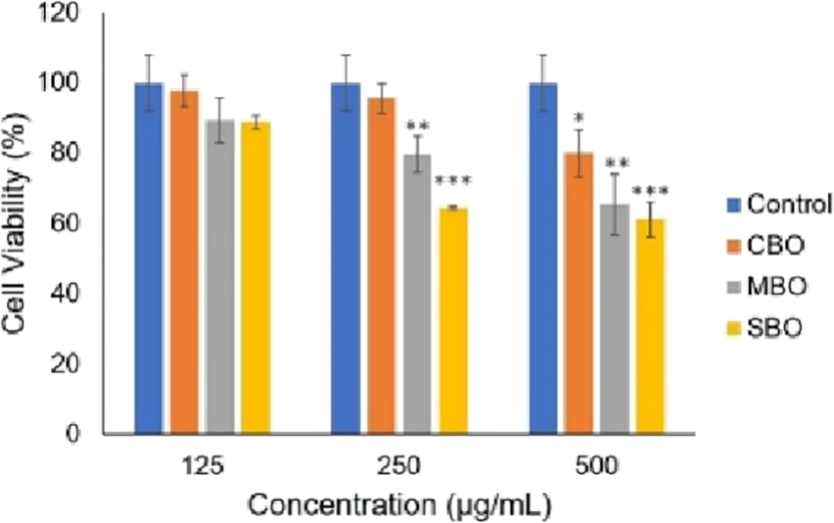
Viability of HUVEC cells after being treated with different particle concentrations for 48 h.

Although direct visualization of cellular uptake was not performed in this study, existing literature on nanoparticle–endothelial cell interactions provides insight into the likely internalization mechanisms. Based on known physicochemical properties of the synthesized particles and the endocytic behavior of HUVECs, it is reasonable to hypothesize that cellular uptake occurs primarily via energy-dependent endocytic pathways.^53^ Specifically, clathrin-mediated and caveolae-mediated endocytoses are the most frequently reported routes for the uptake of nanoscale materials by endothelial cells. Macropinocytosis may also contribute, particularly for submicrometer particles or in response to particle agglomeration.

The submicrometer size, surface characteristics, and potential for receptor-mediated interactions likely facilitate the internalization of these tetraborate particles. It is well established that cellular uptake efficiency and pathway selection are influenced by several nanoparticle properties, including size, surface charge, and surface chemistry.^54^ Given that the particles used in this study meet these criteria, their successful and biocompatible internalization is further supported by the MTT results, which showed no significant cytotoxicity up to 500 μg/mL. These findings support the potential of these materials for safe interaction with endothelial cells and warrant further investigation using advanced imaging and labeling techniques to confirm intracellular localization and trafficking.

## Conclusions

Yb^3+^/Tb^3+^ codoped CaB_4_O_7_ (CBO), MgB_4_O_7_ (MBO), and SrB_4_O_7_ (SBO) were successfully synthesized by three different routes: solid-state (SS), combustion (C), and solution combustion (SC) methods. X-ray diffraction analysis confirmed the phase purity and crystal structures of the materials, with CBO exhibiting a monoclinic structure (space group *P*2_1_/*n*), while MBO and SBO crystallized in orthorhombic phases with space groups *Pcba* and *Pnm*2_1_, respectively. Among the methods, SC and C emerged as the most effective in achieving high phase purity and minimizing secondary phase formation. Crystallite sizes, estimated from XRD, and particle morphology from SEM revealed that MBO produced the smallest and most uniform particles (as small as 150 nm) with minimal agglomeration, especially when synthesized via the C and SC methods.

Upconversion emission measurements demonstrated that codoping with Yb^3+^ and Tb^3+^ yields efficient NIR-excited visible emission, with optimal luminescence observed at 5% Yb^3+^ and 5% Tb^3+^ concentrations. The energy transfer mechanism was attributed to cooperative energy transfer between Yb^3+^ and Tb^3+^ ions, as supported by the absence of upconversion signals in singly doped Tb^3+^ samples under 980 nm excitation. Potential concentration quenching mechanisms, including cross-relaxation between Tb^3+^ ions and Yb–Yb energy migration, were discussed, particularly at higher dopant levels.

Elemental mapping and EDX analyses confirmed uniform dopant distribution in all codoped samples, with no evidence of elemental segregation or contamination. Neutron activation measurements further demonstrated the high thermal neutron absorption capacity of these tetraborates, especially CBO and MBO, with macroscopic cross-sectional values significantly higher than those of conventional ceramics. Their high boron content, phase stability, biocompatibility, and luminescence properties underscore their potential for many applications.

Overall, the structural, optical, and neutron interaction characteristics of Yb^3+^/Tb^3+^ codoped borates position them as promising multifunctional materials for future use in BNCT, photodynamic therapy, biomedical imaging, neutron shielding, and other optoelectronic applications.

## Supplementary Material



## Data Availability

All relevant data are within the manuscript and Supporting Information. Any additional data are available on reasonable request.

## Notes

Additional figures referred as Figures S1–S10 within the main text and their details on XRD data, upconversion, and quantum yield measurements (PDF)
