# Supplementary material for: Structural, Optical, and Neutron Sensitivity Properties of Yb3+/Tb3+ Codoped Alkaline Earth Tetraborates
Source: ACS Omega. 2025 Sep 10;10(37):42589–98. doi: 10.1021/acsomega.5c04342 (PMC12461423; doi:10.1021/acsomega.5c04342)
Supplement: Supplementary file 1 [file ao5c04342_si_001.pdf]

Supplementary Materials

Structural, Optical, and Neutron Sensitivity Properties of Yb^{3+}/Tb^{3+} Co-Doped Alkaline Earth Tetraborates

Özde Ceren Hızal*[a], Okan Esenturk*[a,b], Damla Çetin Altındal[c], Ayşen Yılmaz*[a,b]

[a]*Middle East Technical University, Department of Chemistry, 06800, Ankara, Turkey*
[b]*Middle East Technical University, Dept. of Micro and Nano Tech., 06800, Ankara, Turkey*
[c]*Hacettepe University, Department of Bioengineering, Beytepe, 06800, Ankara, Turkey*
(ceren.hizal@metu.edu.tr, eokan@metu.edu.tr, ayseny@metu.edu.tr)

XRD patterns:

Figures S1-S3 shows the XRD patterns of CBO, MBO, and SBO synthesized with SS, SC, and C methods with their corresponding JCPDS cards.

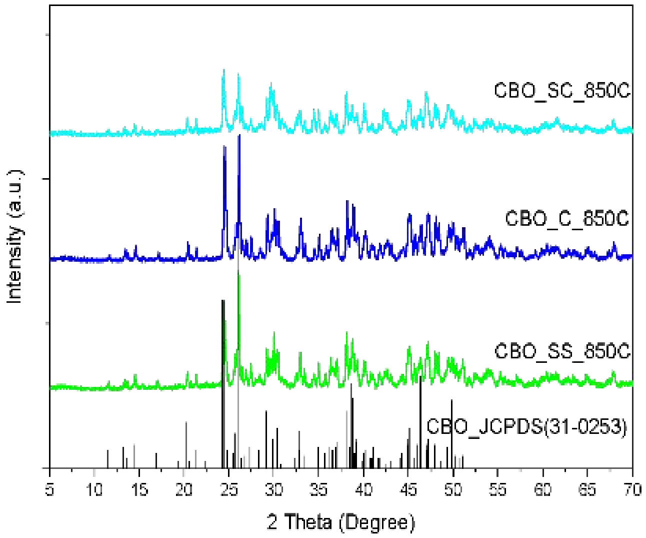

Figure S1. XRD patterns of pure CBO synthesized via SS, SC, and C synthesis.

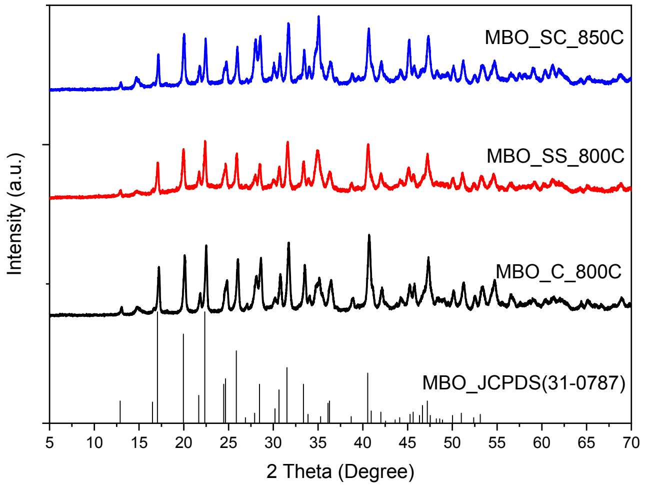

Figure S2. XRD patterns of pure MBO synthesized via SS, SC, and C synthesis.

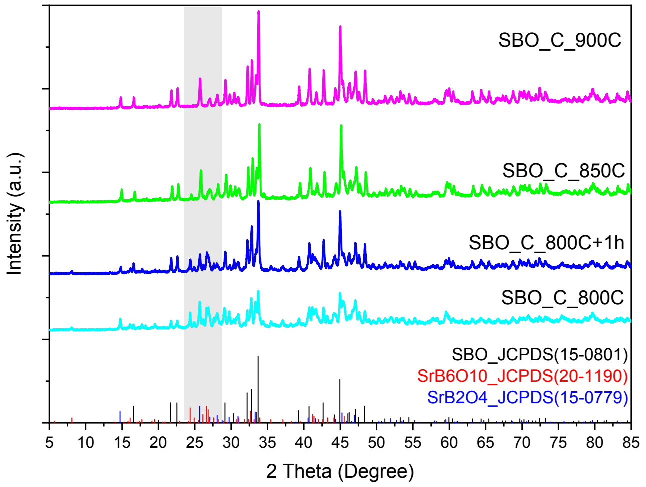

Figure S3. XRD patterns of pure SBO synthesized via SS, SC, and C synthesis.

Upconversion:

As shown in Figure S4, no emission was observed from the singly Tb^{3+} doped and pure tetraborates under 980 nm excitation. To investigate the effect of the synthesis method on the optical properties, Yb^{3+}/Tb^{3+} co-doped tetraborates with the specified concentrations were synthesized using three different methods. While all tetraborates synthesized via the SC and C methods exhibited significant upconversion emissions, those prepared using the SS method did not show any notable emission (Figure S5- S7). Therefore, Tb^{3+} singly doped borates were not synthesized using the SS method.

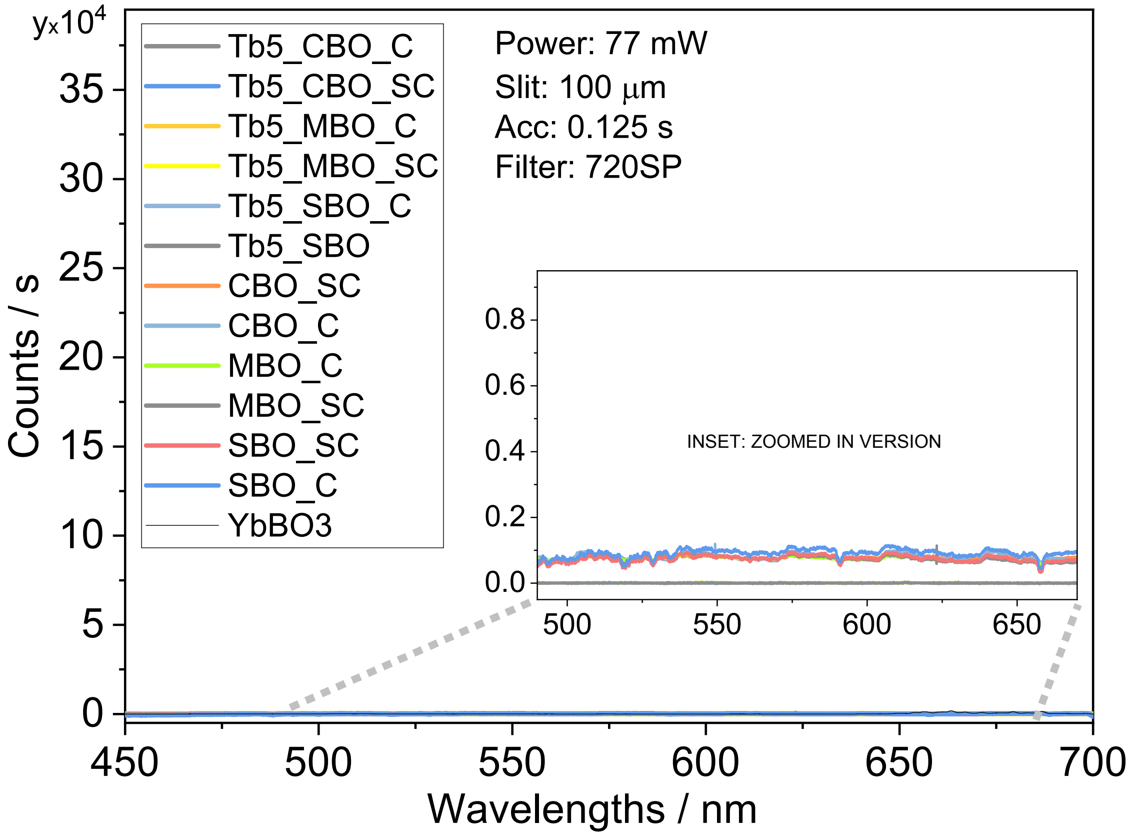

Figure S4. $YbBO_3$, Pure and Tb^{3+} doped CBO, MBO, and SBO products obtained via SC and C method at 980 nm excitation with a laser power of 77mW and power density of 25 mW/ mm^2.

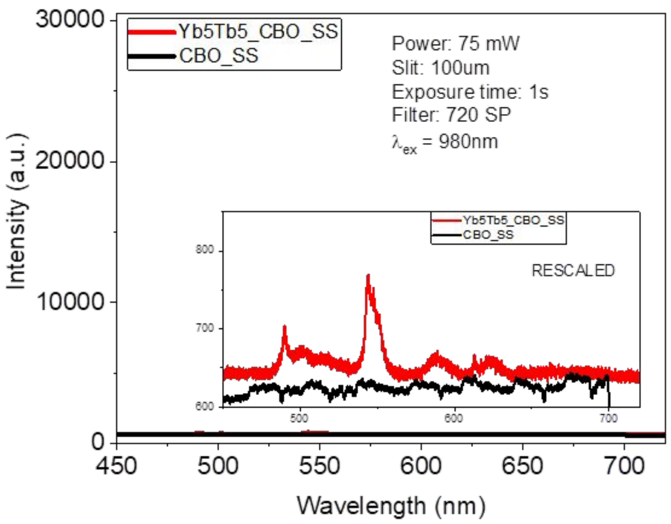

Figure S5. Up-conversion emission spectra of Yb^{3+}/ Tb^{3+} co-doped CBO via SS method at 980 nm excitation with a power density of ~25 mW/mm^2.

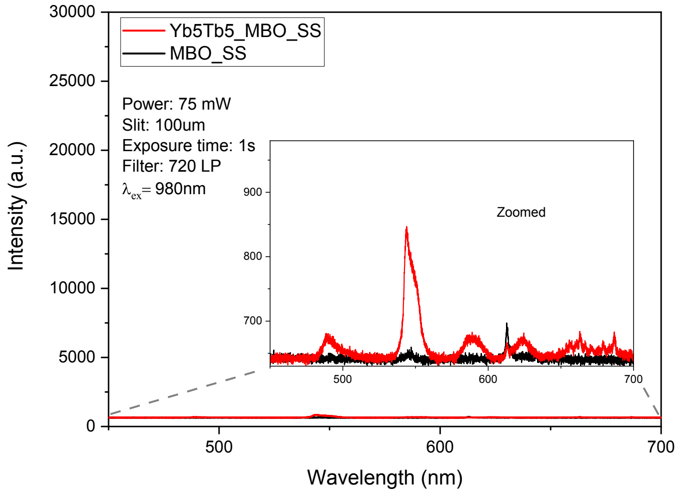

Figure S6. Up-conversion emission spectra of Yb^{3+}/ Tb^{3+} co-doped MBO via SS method at 980 nm excitation with a power density of ~25 mW/mm^2.

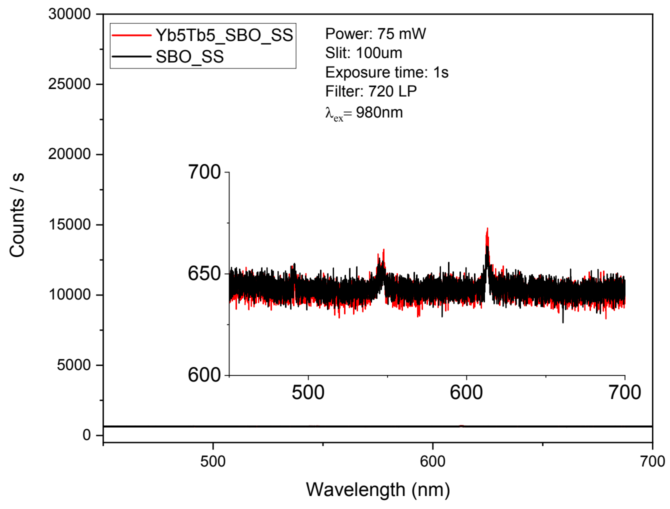

Figure S7. Up-conversion emission spectra of Yb^{3+}/ Tb^{3+} co-doped SBO via SS method at 980 nm excitation with a power density of ~25 mW/mm^2.

Quantum Yield Measurements

Figure S8-S10 shows the quantum yield measurements of selected Yb^{3+}/ Tb^{3+} co-doped tetraborates. The strong emissions are from the sample with laser on and the weak emissions are the instrument response with no laser.

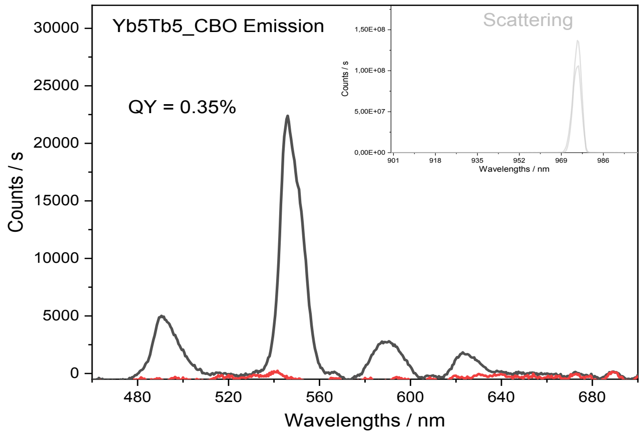

Figure S8. QY measurements of Yb^{3+}/ Tb^{3+} co-doped CBO crystals obtained by SC method excitation with a power density of ~15 mW/ mm^2.

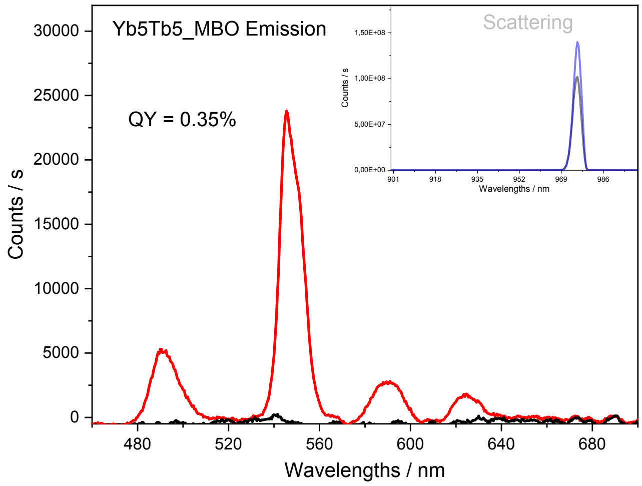

Figure S9. QY measurements of Yb^{3+}/ Tb^{3+} co-doped MBO crystals obtained by C method excitation with a power density of ~15 mW/ mm^2.

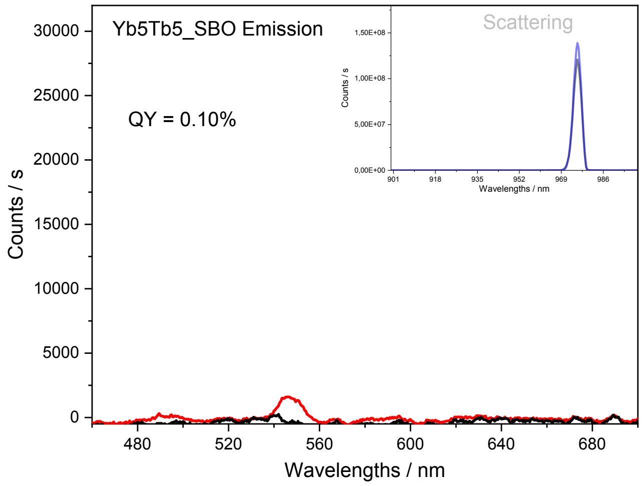

Figure S10. QY measurements of Yb^{3+}/ Tb^{3+} co-doped MBO crystals obtained by SC method excitation with a power density of ~15 mW/mm^2
